# Mandala-based art interventions for anxiety and depressive symptoms in middle-aged and older adults: a systematic review and meta-analysis

**DOI:** 10.3389/fpsyg.2026.1877910

**Published:** 2026-07-01

**Authors:** Linzi Liu, Haiming Hu, Jing Xu, Ye Liu

**Affiliations:** 1 Department of Convergence and Art, Silla University, Busan, Republic of Korea; 2 School of Laboratory Medicine, Hubei University of Chinese Medicine, Wuhan, China

**Keywords:** anxiety, art therapy, depression, mandala coloring, mandala drawing, middle-aged adults, older adults, systematic review

## Abstract

Mandala-based art interventions, which encompass structured coloring as well as open-ended drawing or painting, have gained increasing recognition as low-cost, non-pharmacological strategies for emotional regulation. Nevertheless, empirical evidence regarding their effectiveness, particularly among middle-aged and older adults, remains fragmented. To address this gap, the present systematic review and meta-analysis synthesized randomized controlled trials that evaluated the effects of mandala-based art interventions on anxiety and depressive symptoms in individuals aged 55 years or older. A comprehensive literature search was conducted in PubMed, Web of Science, Embase, SinoMed, and Wanfang from database inception to May 1, 2026. Two independent reviewers screened records, extracted data, and assessed risk of bias using the Cochrane risk-of-bias tool version 2. Pooled standardized mean differences (SMDs; Hedges’ *g*) with 95% confidence intervals (CI) were obtained using random-effects models. Nine randomized controlled trials comprising 648 participants met the inclusion criteria. Relative to control conditions, mandala-based art interventions were associated with significantly lower anxiety symptoms (SMD = −0.95, 95% CI: −1.43 to −0.48, *p* < 0.0001; *I*^2^ = 85%) and lower depressive symptoms (SMD = −1.21, 95% CI: −1.86 to −0.56, *p* = 0.0002; *I*^2^ = 84%), although heterogeneity was substantial for both outcomes. Exploratory subgroup analyses by intervention frequency and mandala format were conducted descriptively. These subgroup findings should be interpreted as hypothesis-generating only because of the small number of studies, substantial heterogeneity, and absence of direct randomized comparisons. Sensitivity analyses did not change the direction of the pooled effects, but substantial heterogeneity persisted. The certainty of evidence was very low for both outcomes because of risk of bias, inconsistency, indirectness, imprecision, and the small number of trials. Therefore, the findings should be interpreted as preliminary rather than definitive evidence of clinical effectiveness. More rigorously designed, adequately powered, and transparently reported randomized trials are necessary before definitive clinical recommendations can be formulated.

**Systematic review registration:**

PROSPERO, CRD420261389239.

## Graphical abstract

**Figure:**
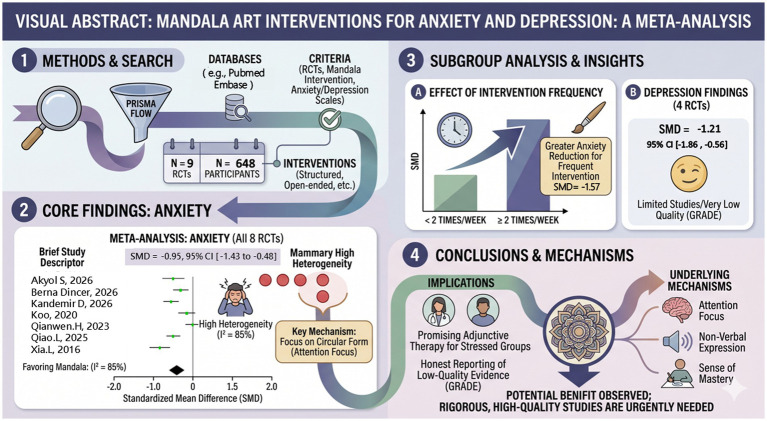

## 1 Introduction

Anxiety and depressive symptoms are common psychological concerns among middle-aged and older adults. This is particularly true for individuals living with chronic illness, those receiving long-term institutional care, and patients undergoing major medical procedures (Balsamo et al., 2018; World Health Organization, 2025). Such symptoms may compromise quality of life, disrupt sleep patterns, reduce social participation, complicate disease self-management, and escalate the demand for health and care services. Although pharmacological treatments and psychological therapies remain essential components of mental health care, many older adults encounter substantial barriers to accessing formal services. These barriers include limited availability of specialized providers, concerns about medication burden and polypharmacy, and reluctance to engage in verbally demanding psychotherapeutic approaches. Consequently, accessible, acceptable, and low-cost non-pharmacological strategies are needed as complementary avenues for emotional support.

Art-based interventions have been widely implemented in health, community, and long-term care settings. One key advantage is that they enable participants to express emotions through visual and sensory processes rather than relying exclusively on verbal communication (Stuckey and Nobel, 2010; Fancourt and Finn, 2019). Among these creative modalities, mandala-based art interventions represent a distinct category of activity typically characterized by circular, symmetrical, or center-oriented patterns. These interventions can be delivered in two primary formats: structured mandala coloring, in which participants color pre-designed patterns, and open-ended mandala drawing or painting, in which participants generate their own designs from a blank template. The structured and repetitive visual features of mandalas may promote attentional focus, emotional containment, relaxation, and a sense of order. In contrast, open-ended mandala creation may offer greater latitude for personal expression and meaning-making (Curry and Kasser, 2005; Henderson et al., 2007).

Several randomized controlled trials have examined the emotional effects of mandala-based interventions across diverse populations. These include community-dwelling older adults, institutionalized older adults, and clinical groups such as patients with chronic heart failure, cancer, stroke, or perioperative conditions (Akyol and Demir Yildirim, 2026; Dincer et al., 2026; Qianwen et al., 2025; Kandemir et al., 2026; Koo et al., 2020; Xia and Jinwen, 2018; Qiao et al., 2025; Haiying et al., 2021; Shufen et al., 2019). However, individual trials have generally been small-scale and varied considerably in participant characteristics, intervention format, frequency, duration, control conditions, and outcome measures. Such heterogeneity complicates efforts to determine whether mandala-based interventions consistently reduce anxiety or depressive symptoms and whether specific intervention characteristics are associated with larger effect magnitudes.

To address this knowledge gap, the present systematic review and meta-analysis synthesized randomized controlled trials examining mandala-based art interventions for anxiety and depressive symptoms in middle-aged and older adults. The investigation aimed to estimate the overall effects of these interventions, explore whether intervention frequency and mandala format were associated with variation in outcomes, and evaluate the robustness and certainty of the available evidence.

## 2 Methods

### 2.1 Study design and reporting guideline

This systematic review and meta-analysis were conducted in accordance with the Preferred Reporting Items for Systematic Reviews and Meta-Analyses (PRISMA) 2020 statement (Page et al., 2021). The protocol was registered in the Prospective Register of Systematic Reviews (PROSPERO; registration number: CRD420261389239). The review focused exclusively on randomized controlled trials of mandala-based art interventions in adults aged 55 years or older.

### 2.2 Eligibility criteria

Eligibility criteria were defined using the PICOS framework. Studies were included if they met all of the following conditions: (1) a randomized controlled trial design; (2) participants aged 55 years or older; (3) an intervention consisting of mandala coloring, mandala drawing, mandala painting, or any clearly mandala-based art activity; (4) a control condition involving usual care, no intervention, reading, health education, psychological support, or another non-art control; and (5) at least one validated measure of anxiety or depressive symptoms. Exclusion criteria were as follows: interventions not based on mandala activities; mandala activities combined with another active therapy in a way that precluded isolation of the mandala effect; non-randomized study designs; unavailable outcome data; and publication types such as conference abstracts, dissertations, reviews, case reports, or duplicate reports.

### 2.3 Information sources and search strategy

PubMed, Web of Science, Embase, SinoMed, and Wanfang were searched from inception to May 1, 2026. The search strategy combined controlled vocabulary and free-text terms covering mandala-based art activities, anxiety and depressive symptoms, middle-aged or older adults, and randomized or controlled trial designs. Broad art-related terms were retained to improve search sensitivity, but all retrieved records were screened strictly against the eligibility criterion that the intervention had to be clearly mandala-based. The RCT-related terms were used as a pragmatic trial-design search block rather than as a formally validated RCT filter. To address the possibility that trial-design terms may have reduced search sensitivity, supplementary searches without the RCT-related search block were also conducted. These supplementary searches identified additional records for screening but did not identify additional eligible randomized controlled trials. Reference lists of included studies were also screened. The complete search strategies for each database, including English and Chinese search terms and supplementary searches without the RCT-related search block, are provided in Supplementary Table S1.

### 2.4 Study selection

All retrieved records were imported into EndNote X9, and duplicate entries were removed. Two independent reviewers screened titles and abstracts against the eligibility criteria. Full texts of potentially eligible studies were then obtained and assessed independently by the same reviewers. Disagreements were resolved through discussion; when consensus could not be reached, a third reviewer was consulted. The study selection process is summarized in the PRISMA flow diagram.

### 2.5 Data extraction

Two reviewers independently extracted data using a standardized data extraction form. Extracted information included first author, publication year, country or region (when available), participant characteristics, sample size, age, sex distribution, health status, mandala format, intervention frequency, intervention duration, control condition, outcome measures, and quantitative data required for meta-analysis. Any disagreements were resolved by discussion or consultation with a third reviewer. For quantitative synthesis, post-intervention means, standard deviations, and sample sizes were preferentially extracted for each eligible outcome; when these were unavailable, change-score data or convertible statistics were extracted where possible.

### 2.6 Risk-of-bias assessment

Risk of bias was assessed using the Cochrane risk-of-bias tool version 2 (RoB 2) for randomized trials (Sterne et al., 2019). The assessment covered five domains: bias arising from the randomization process, bias due to deviations from intended interventions, bias due to missing outcome data, bias in outcome measurement, and bias in selection of the reported result. Each domain was rated as low risk of bias, some concerns, or high risk of bias. The overall risk-of-bias judgment was determined according to RoB 2 guidance. Two reviewers independently performed the assessment, and disagreements were resolved through discussion or consultation with a third reviewer.

### 2.7 Certainty of evidence

The certainty of evidence for each primary outcome was evaluated using the Grading of Recommendations Assessment, Development and Evaluation (GRADE) approach (Guyatt et al., 2008). Certainty was rated as high, moderate, low, or very low based on five domains: risk of bias, inconsistency, indirectness, imprecision, and publication bias. Because fewer than 10 studies were available for each outcome, formal tests for small-study effects or publication bias were not conducted; instead, publication bias was considered qualitatively.

### 2.8 Statistical analysis

RevMan 5.4 was used to generate forest plots and conduct subgroup analyses, and Stata 14.0 was used for sensitivity analyses. Because anxiety and depressive symptoms were measured with different scales across studies, standardized mean differences (SMDs; Hedges’ *g*) with 95% confidence intervals (CI) were used as effect sizes. Hedges’ g was used because it incorporates a small-sample correction, which was appropriate given the small sample sizes of several included trials. For the primary meta-analysis, post-intervention mean scores were used whenever available; change scores were used only when post-intervention values were unavailable. Means, standard deviations, and sample sizes for intervention and control groups were extracted directly from the included studies whenever possible. When standard deviations were not directly reported, they were calculated from other available statistics, such as standard errors, confidence intervals, *p* values, or test statistics, where sufficient information was provided. A negative SMD indicated greater reductions in anxiety or depressive symptoms in the mandala intervention group compared with the control group. Given the anticipated clinical and methodological diversity across participant populations, intervention formats, durations, and outcome measures, random-effects models were adopted *a priori*. Pooling was considered appropriate for estimating a broad average effect because all included studies were randomized controlled trials of clearly mandala-based activities and assessed anxiety-related or depressive symptoms using validated scales. However, the pooled estimates were not intended to represent precise intervention effects for any single clinical population, setting, or mandala protocol. Statistical heterogeneity was assessed using the *I*^2^ statistic (Higgins et al., 2003), with *I*^2^ values of 50% or higher considered indicative of substantial heterogeneity. Exploratory subgroup analyses were conducted according to intervention frequency and mandala format. Leave-one-out sensitivity analyses were used to evaluate whether a single study drove the pooled results. Because subgroup analyses involved very few studies per subgroup, they are hypothesis-generating only. No direct statistical comparisons between subgroups were performed, and any observed numerical differences should not be interpreted as evidence of superiority of one intervention format or frequency over another. Accordingly, subgroup analyses were used only to describe whether effect estimates varied across categories, not to infer dose–response relationships or comparative effectiveness. When multiple post-intervention time points were available, the outcome measured immediately after completion of the intervention was extracted. For all included scales, higher scores indicated more severe anxiety or depressive symptoms; therefore, negative SMD consistently favored mandala-based art interventions. When multiple eligible scales were reported for the same outcome, the scale most directly corresponding to anxiety or depressive symptoms was prioritized.

## 3 Results

### 3.1 Study selection

**Figure 1:**
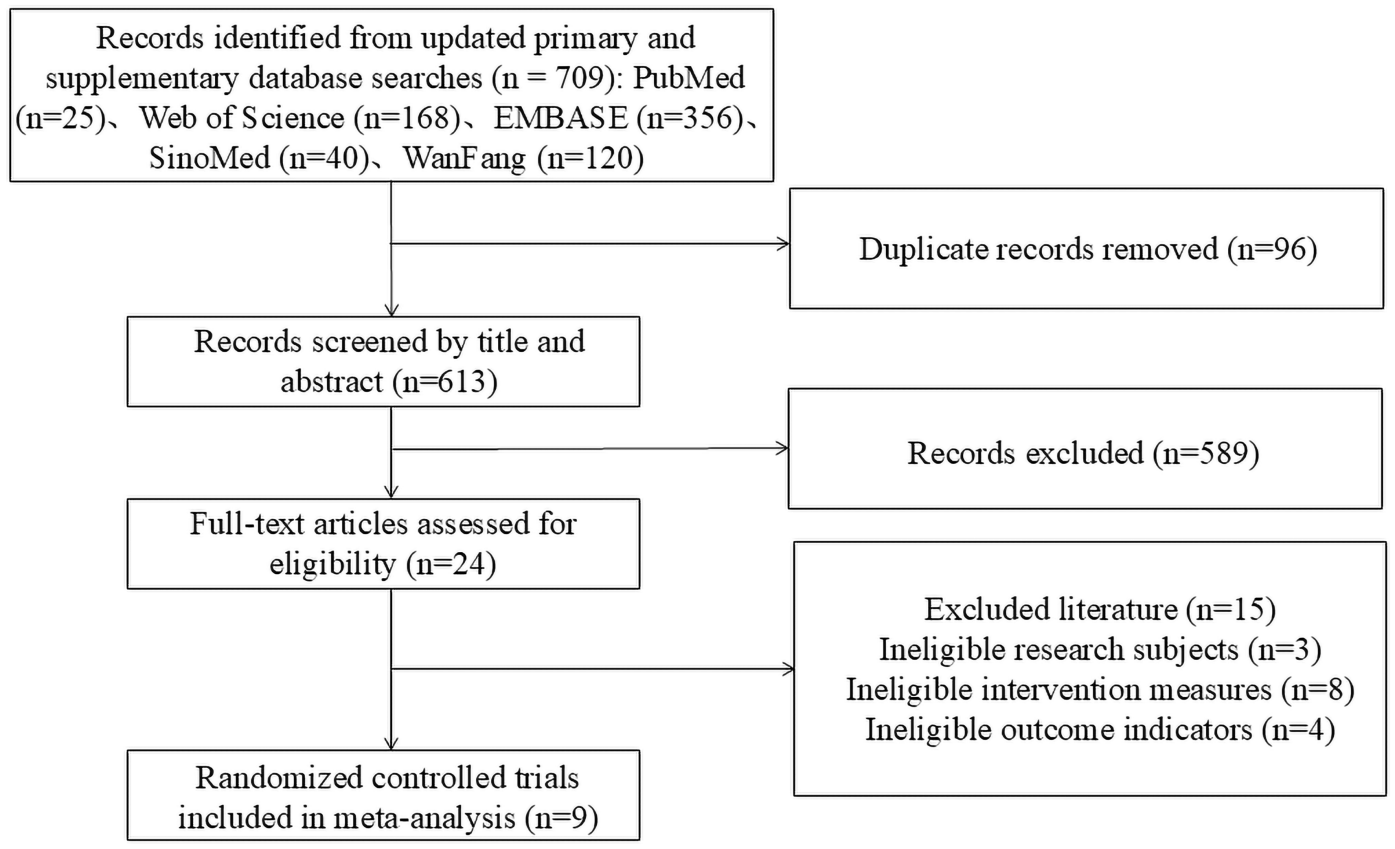
PRISMA flow diagram of study identification, screening, eligibility assessment, and inclusion.

The updated primary and supplementary database searches yielded 709 records. This total reflected the final search set used for screening, including the expanded database searches and supplementary searches conducted without the RCT-related search block. After removal of duplicates, 613 records remained for title and abstract screening. A total of 589 records were excluded as clearly irrelevant. Of the 24 full-text articles assessed for eligibility, 15 were excluded because of ineligible research subjects (*n* = 3), ineligible intervention measures (*n* = 8), or ineligible outcome indicators (*n* = 4). Consequently, nine randomized controlled trials were included in the systematic review and meta-analysis (Figure 1).

### 3.2 Study characteristics

The nine included trials involved 648 participants and were published between 2018 and 2026 (Akyol and Demir Yildirim, 2026; Dincer et al., 2026; Qianwen et al., 2025; Kandemir et al., 2026; Koo et al., 2020; Xia and Jinwen, 2018; Qiao et al., 2025; Haiying et al., 2021; Shufen et al., 2019). Study populations encompassed community-dwelling middle-aged or older adults, residents of nursing or integrated care facilities, and clinical populations with chronic heart failure, coronary angiography, lung adenocarcinoma undergoing chemotherapy, post-stroke depression, esophageal cancer, or postoperative gynecological cancer. Mandala formats included structured coloring and open-ended drawing or painting. Intervention frequency ranged from a single session to daily or multiple weekly sessions, and intervention duration ranged from a single session to 12 weeks. Control conditions comprised usual care, no intervention, reading, and routine health education or psychological support. The main outcome measures included the State–Trait Anxiety Inventory (STAI), Self-Rating Anxiety Scale (SAS), Hamilton Anxiety Rating Scale (HAMA), Hamilton Depression Rating Scale (HAMD), Self-Rating Depression Scale (SDS), Geriatric Depression Scale (GDS), and Death Anxiety Scale (DAS) (Table 1).

**Table 1:** Characteristics of included randomized controlled trials.

Study	Participants/setting	Sample size, *n* (I/C)	Age, years (I/C)	Sex distribution (male/female, I/C)	Mandala format	Frequency and duration	Comparator	Outcome(s)
Dincer et al. (2026)	Nursing-home residents; cognitively intact; no diagnosed psychiatric disorder	93 (I/C: NR)	76.45 ± 8.52/73.18 ± 7.15	47/53/45/55	Structured mandala coloring	3 sessions/week for 12 weeks	Daily routine without additional intervention	DAS
Koo et al. (2020)	Community-dwelling older adults; not using anxiolytic or antidepressant medication	60 (30/30)	65.4 ± 6.4/64.7 ± 5.3	4/26/9/21	Structured mandala coloring	Single 20-min session	Reading ordinary material for 20 min	STAI
Qianwen et al. (2025)	Older adults with stable chronic heart failure, NYHA class II–III	80 (40/40)	76.03 ± 6.03/73.33 ± 6.75	20/20/21/19	Structured mandala coloring	5 sessions/week for 2 weeks	Routine treatment and nursing care	SAS
Haiying et al. (2021)	Older adults newly admitted to an integrated medical-care center	57 (35/22)	≥65 years/≥65 years	13/22/1/21	Structured mandala coloring	2 sessions/week for 8 weeks	Routine nursing care	GDS
Kandemir et al. (2026)	Patients undergoing coronary angiography	90 (45/45)	57.88 ± 11.45/59.80 ± 5.14	33/12/30/15	Structured mandala coloring	Single session	Routine care	STAI
Qiao et al. (2025)	Patients with stage IIIB or IV lung adenocarcinoma receiving chemotherapy	106 (53/53)	56.89 ± 5.56/57.62 ± 5.27	28/25/30/23	Open-ended mandala drawing/painting	3 sessions/week for 4 weeks	Routine intervention, health education, and psychological counseling	HAMD; HAMA
Xia and Jinwen (2018)	Patients with post-stroke depression	60 (I/C: NR)	Approximately 60 years	NR	Open-ended mandala drawing/painting	2 sessions/week for 8 weeks	Routine treatment and nursing care	HAMD; HAMA
Shufen et al. (2019)	Patients with esophageal cancer	60 (I/C: NR)	Approximately 60 years	NR	Structured mandala coloring	1 session/week for 6 weeks	Routine nursing care	SDS
Akyol and Demir Yildirim (2026)	Postoperative patients with gynecological cancer	42 (21/21)	57.6 ± 10.5/58.2 ± 12.1	0/21/0/21	Structured mandala coloring	Daily for 3 days	Routine care	STAI

I, intervention group; C, control group; NR, not reported; NYHA, New York Heart Association. Age is reported as mean ± SD unless otherwise stated. Sex distribution is reported as male/female for the intervention and control groups where available. DAS, Death Anxiety Scale; GDS, Geriatric Depression Scale; HAMA, Hamilton Anxiety Rating Scale; HAMD, Hamilton Depression Rating Scale; SAS, Self-Rating Anxiety Scale; SDS, Self-Rating Depression Scale; STAI, State–Trait Anxiety Inventory.

### 3.3 Risk of bias

**Figure 2:**
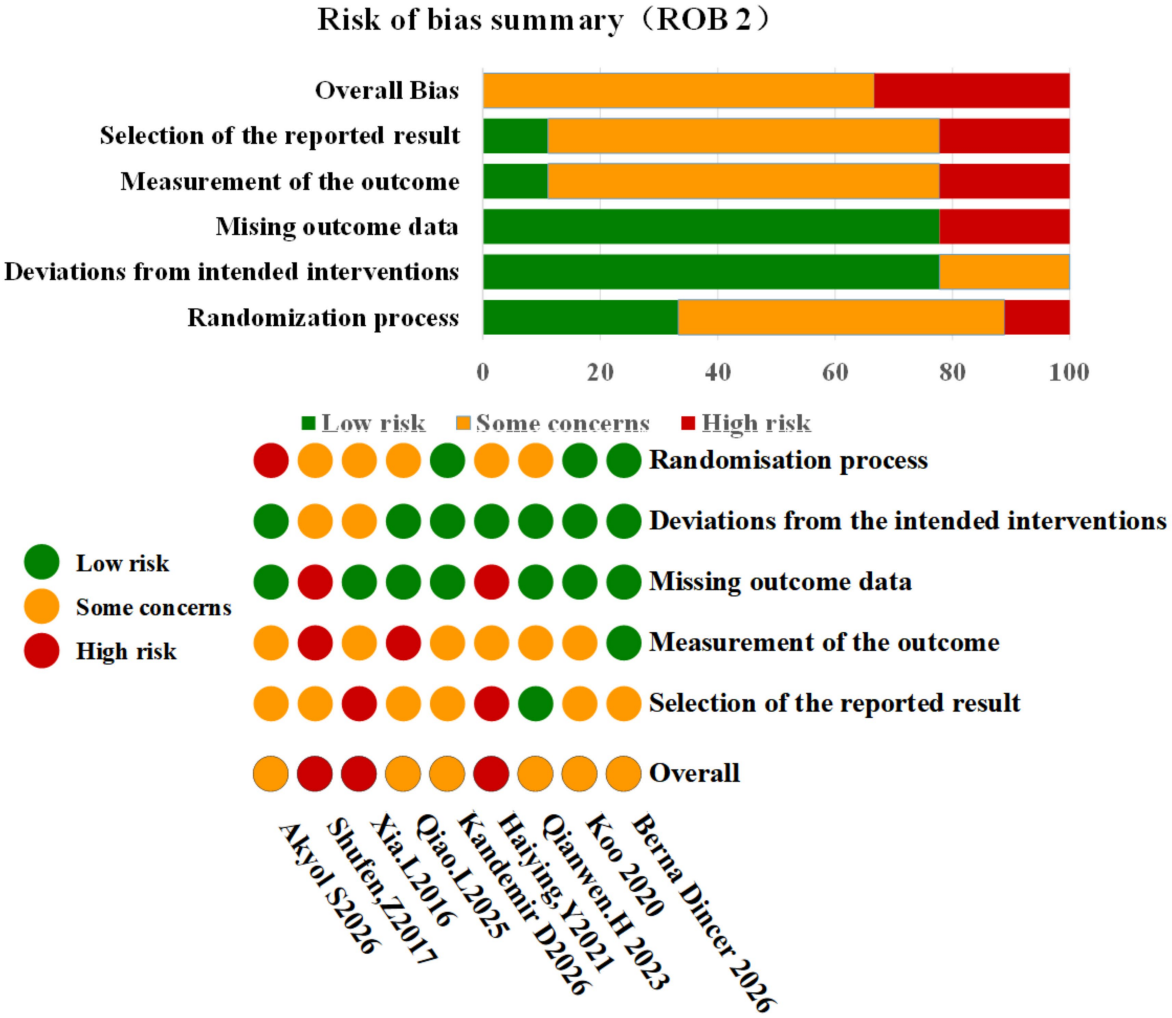
Summary of risk-of-bias assessment using the Cochrane RoB 2 tool.

Using the RoB 2 tool, six studies (Dincer et al., 2026; Koo et al., 2020; Qianwen et al., 2025; Haiying et al., 2021; Qiao et al., 2025; Akyol and Demir Yildirim, 2026) were judged as having some concerns, and three studies (Kandemir et al., 2026; Xia and Jinwen, 2018; Shufen et al., 2019) were judged as having a high risk of bias. The most common concerns include insufficient detail about the randomization process, limited information on allocation concealment or deviations from intended interventions, and potential bias in outcome measurement or selective reporting. A summary of risk-of-bias judgments is shown in Figure 2. Given the predominance of studies with concerns or a high risk of bias, the pooled effect estimates should be interpreted cautiously, as methodological limitations may have influenced the magnitude of the observed effects.

### 3.4 Effects on anxiety symptoms

**Figure 3:**
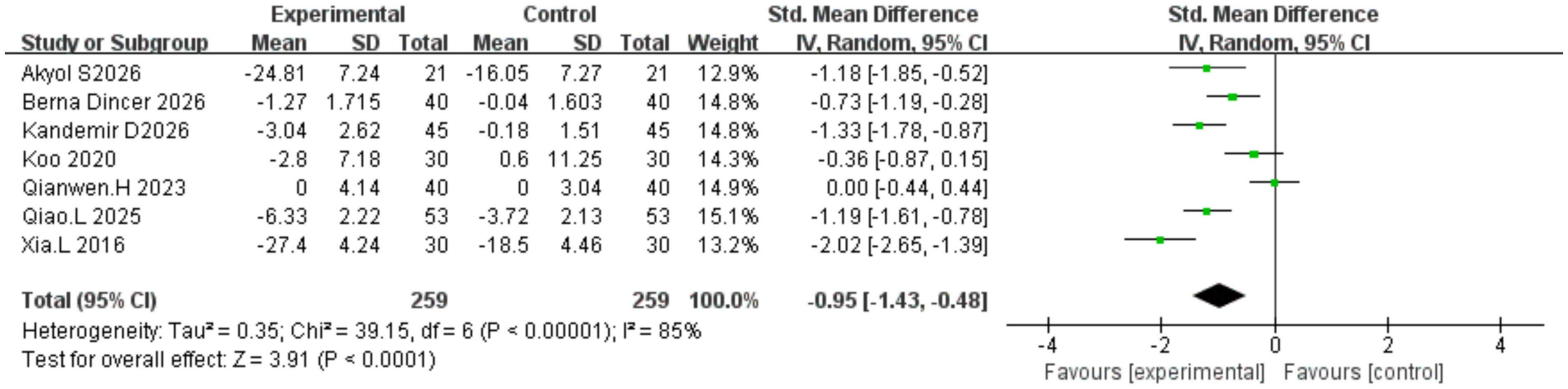
Forest plot of the effects of mandala-based art interventions on anxiety symptoms. Effect estimates are presented as standardized mean differences (Hedges’ *g*) with 95% confidence intervals.

Seven studies evaluated the effect of mandala-based art interventions on anxiety-related outcomes. Substantial heterogeneity was observed (*I*^2^ = 85%), and a random-effects model was therefore applied. The pooled analysis showed that mandala-based art interventions were associated with significantly lower anxiety symptoms compared with control conditions (SMD = −0.95, 95% CI: −1.43 to −0.48, *p* < 0.0001; Figure 3). The substantial heterogeneity (*I*^2^ = 85%) indicates considerable variation across studies; this likely reflects differences in clinical populations, intervention characteristics (format, frequency, duration), control conditions, and outcome measures. Therefore, the pooled estimate should be interpreted with appropriate caution. One anxiety-related study assessed death anxiety rather than general anxiety; therefore, the influence of this study was further examined in a *post-hoc* sensitivity analysis.

### 3.5 Subgroup analysis for anxiety symptoms

**Figure 4:**
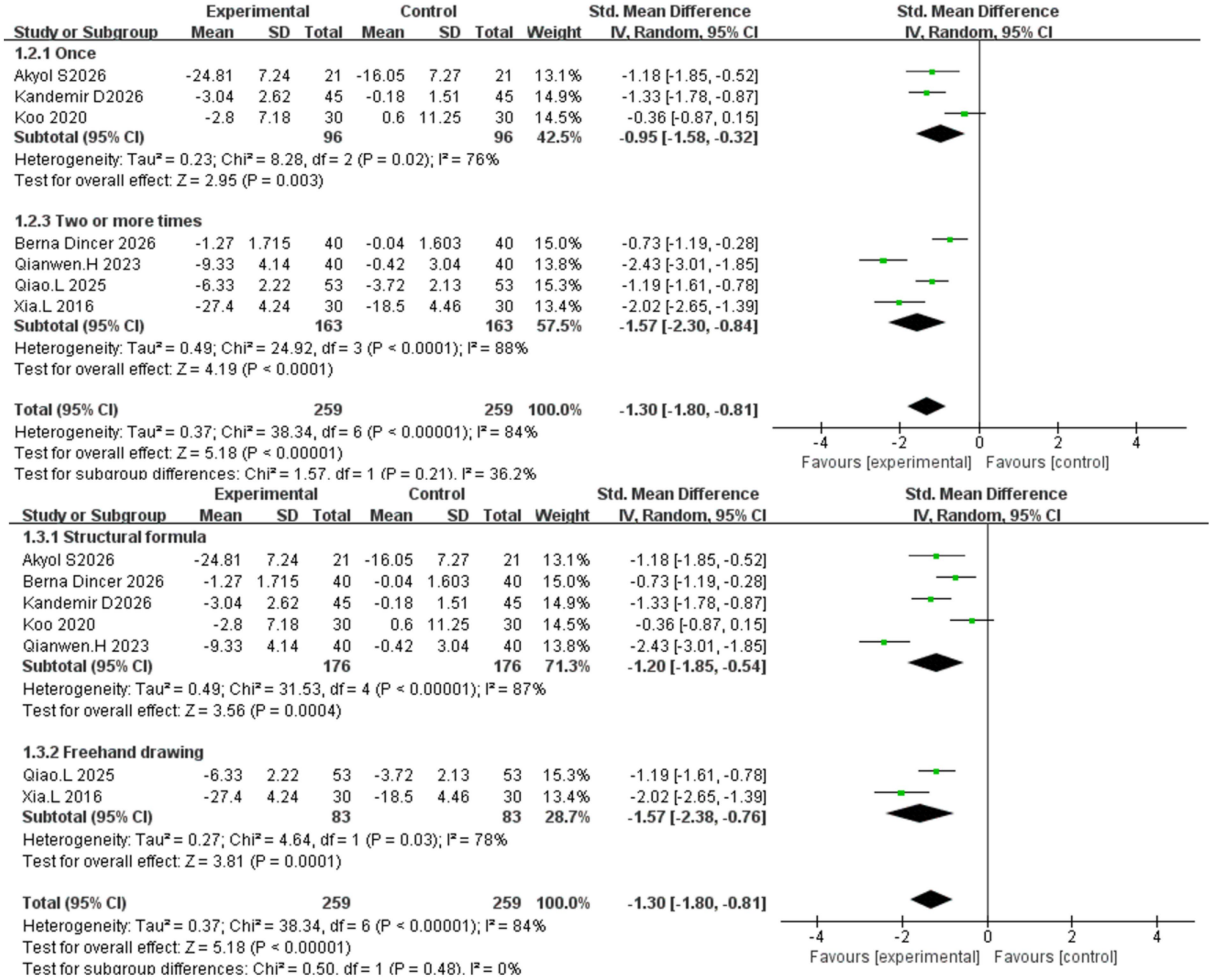
Exploratory subgroup analyses for anxiety symptoms by intervention frequency and mandala format. Subgroup estimates are descriptive and should not be interpreted as evidence of comparative effectiveness.

Exploratory and descriptive subgroup analyses were conducted according to intervention frequency and mandala format. The pooled estimates were below zero for both single-session interventions (SMD = −0.95, 95% CI: −1.58 to −0.32, *I*^2^ = 76%, *p* = 0.003) and interventions delivered two or more times per week (SMD = −1.57, 95% CI: −2.30 to −0.84, *I*^2^ = 88%, *p* < 0.0001), indicating effect estimates favoring mandala-based interventions in both subgroups. Although subgroup estimates appeared to differ numerically, these analyses were based on small numbers of studies, were potentially confounded by population, intervention duration, control condition, and study quality, and did not involve direct head-to-head comparisons; thus, apparent subgroup differences should be interpreted as descriptive and hypothesis-generating rather than confirmatory. The pooled estimates also favored mandala-based interventions in both the structured coloring subgroup (SMD = −1.20, 95% CI: −1.85 to −0.54, *I*^2^ = 87%, *p* = 0.0004) and the open-ended drawing or painting subgroup (SMD = −1.57, 95% CI: −2.38 to −0.76, *I*^2^ = 78%, *p* = 0.0001). The observed numerical differences between subgroups are exploratory and should not be interpreted as evidence that one mandala format or intervention frequency is superior to another; direct randomized comparative trials are needed. Furthermore, the intervention format was confounded with population, duration, and study quality (Figure 4).

### 3.6 Sensitivity analysis for anxiety symptoms

**Figure 5:**
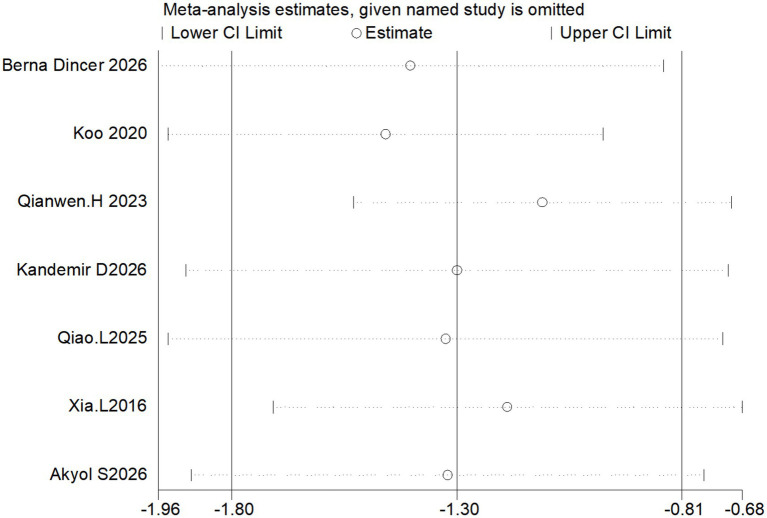
Leave-one-out sensitivity analysis for anxiety symptoms.

Leave-one-out sensitivity analyses indicated that the direction of the pooled effect remained unchanged after omitting each study in turn. The pooled effect remained statistically significant across sensitivity analyses, suggesting that the anxiety finding was not driven by a single study (Figure 5). Nevertheless, substantial heterogeneity persisted, indicating that differences in populations, outcome scales, intervention characteristics, and control conditions likely contributed to between-study variability. In addition, because one study assessed death anxiety rather than general anxiety, a post-hoc sensitivity analysis was performed by excluding the Dincer et al. (2026) study. The pooled effect remained statistically significant for the remaining six studies (SMD = −0.82, 95% CI: −1.33 to −0.31, *p* = 0.002), and heterogeneity was slightly reduced but remained substantial (*I*^2^ = 79%). A further post-hoc sensitivity analysis excluding studies judged as having a high risk of bias yielded a pooled effect of SMD = −0.85, 95% CI: −1.30 to −0.29, *p* = 0.009; *I*^2^ = 69%. The direction of the effect remained unchanged, although the estimate should still be interpreted cautiously because heterogeneity remained substantial and the number of remaining studies was small.

### 3.7 Effects on depressive symptoms

**Figure 6:**
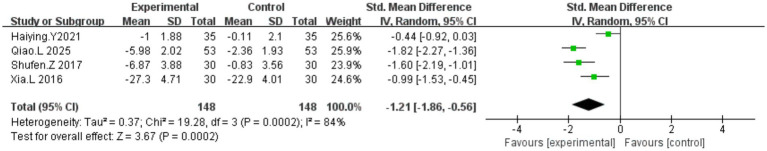
Forest plot of the effects of mandala-based art interventions on depressive symptoms. Effect estimates are presented as standardized mean differences (Hedges’ *g*) with 95% confidence intervals.

Four studies evaluated depressive symptoms. Substantial heterogeneity was observed (*I*^2^ = 84%), and a random-effects model was used. The pooled analysis showed that mandala-based art interventions were associated with significantly lower depressive symptoms compared with control conditions (SMD = −1.21, 95% CI: −1.86 to −0.56, *p* = 0.0002; Figure 6). The substantial heterogeneity (*I*^2^ = 84%) suggests notable between-study variation, probably reflecting differences in participant health status, intervention protocols, and outcome instruments. Therefore, the summary estimate should be interpreted as provisional.

### 3.8 Subgroup analysis for depressive symptoms

**Figure 7:**
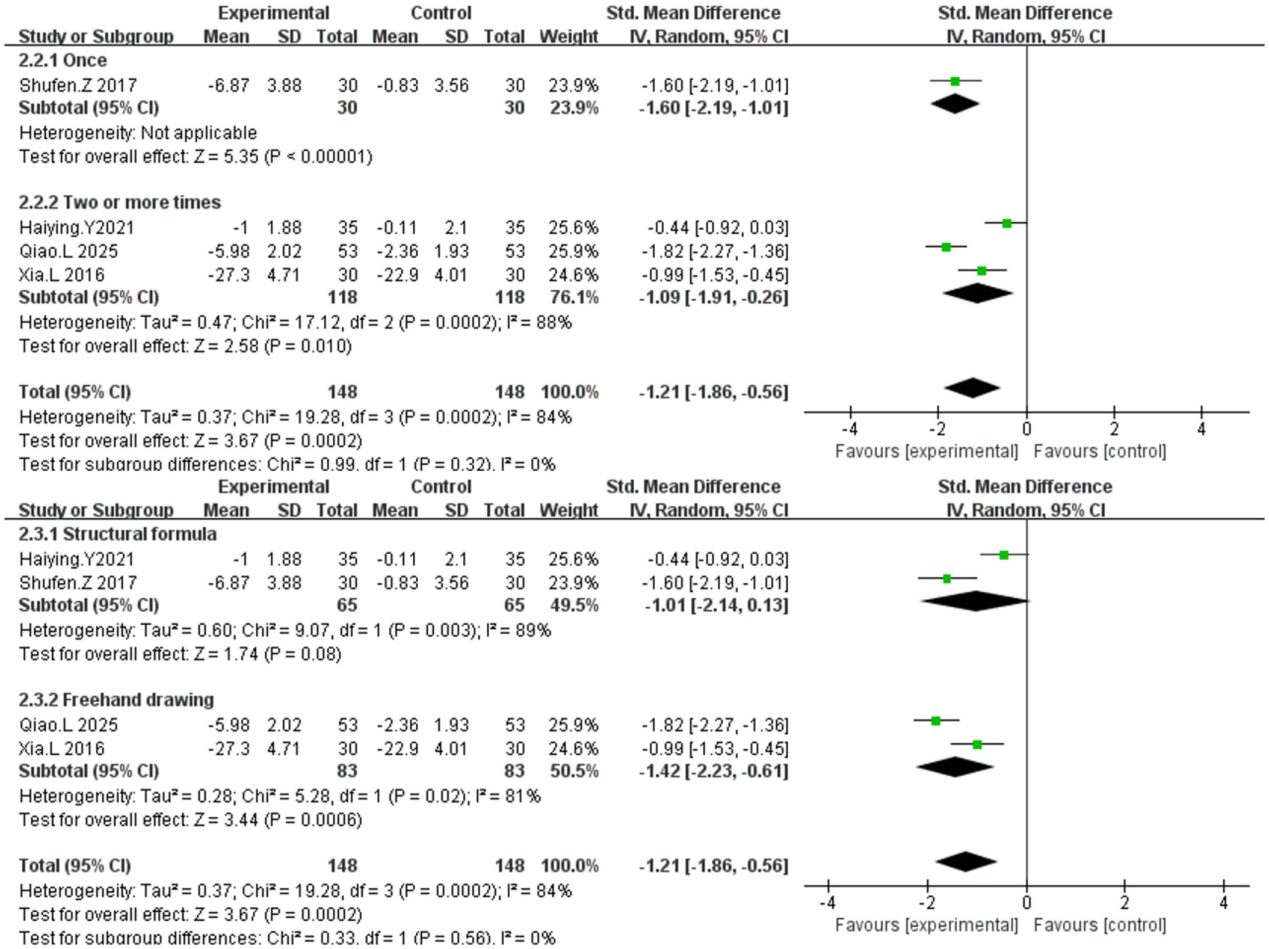
Exploratory subgroup analyses for depressive symptoms by intervention frequency and mandala format. Subgroup estimates are descriptive and should not be interpreted as evidence of comparative effectiveness.

In exploratory and descriptive subgroup analyses by intervention frequency, the pooled estimates favored mandala-based interventions in both once-weekly or single-session interventions (SMD = −1.60, 95% CI: −2.19 to −1.01, *p* < 0.00001) and interventions delivered two or more times per week (SMD = −1.09, 95% CI: −1.91 to −0.26, *I*^2^ = 88%, *p* = 0.01). However, this pattern should be interpreted cautiously because subgroup sizes were very small, clinical and methodological features differed across studies, and no direct randomized comparisons of intervention frequency were available. In the subgroup analysis by mandala format, the pooled estimate for open-ended mandala drawing or painting was below zero (SMD = −1.42, 95% CI: −2.23 to −0.61, *I*^2^ = 89%, *p* = 0.0006), whereas the confidence interval for the structured coloring subgroup crossed the line of no effect (SMD = −1.01, 95% CI: −2.14 to 0.13, *I*^2^ = 91%, *p* = 0.08). These subgroup findings are exploratory and descriptive only and should not be used to infer comparative superiority, dose–response effects, or differential effectiveness of one format or frequency without adequately powered direct randomized comparisons (see Figure 7).

### 3.9 Sensitivity analysis for depressive symptoms

**Figure 8:**
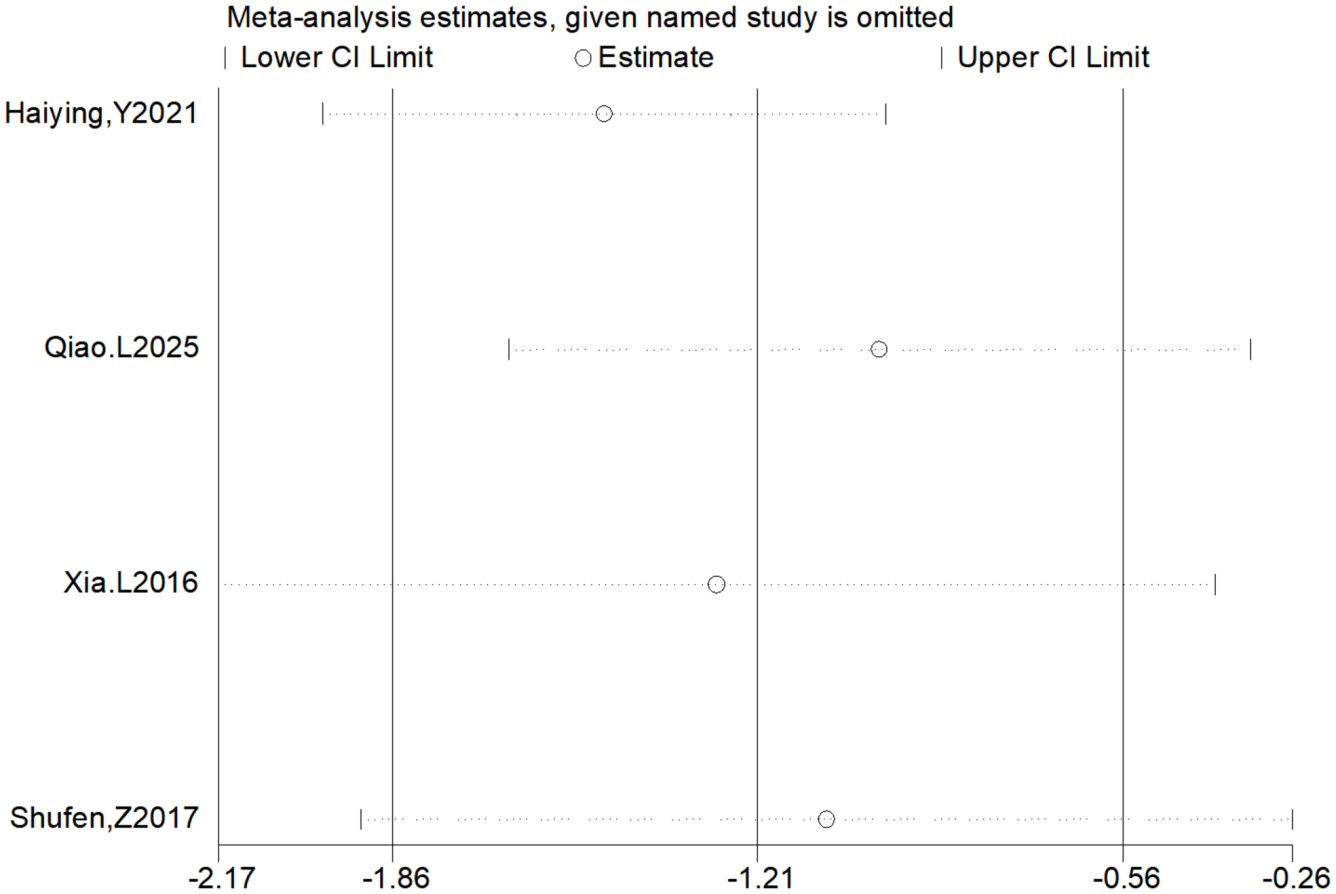
Leave-one-out sensitivity analysis for depressive symptoms.

Leave-one-out sensitivity analyses showed that the direction of the pooled effect remained unchanged after excluding each study in turn, and the pooled effect remained statistically significant. These results suggest that the finding on depressive symptoms was robust despite the small number of studies. However, the high level of heterogeneity limits the precision and generalizability of the pooled estimate (Figure 8). Because only four studies contributed to the depressive symptom analysis, excluding high-risk-of-bias studies left too few studies for a stable pooled estimate. Therefore, the influence of study quality on the depressive symptom finding could not be fully resolved, and this uncertainty was incorporated into the GRADE assessment.

### 3.10 Certainty of evidence

The certainty of evidence was downgraded due to risk-of-bias concerns, substantial inconsistency, indirectness, and imprecision stemming from the small number of studies and limited sample sizes. Publication bias could not be formally assessed because fewer than 10 studies were available for each outcome. Indirectness was rated as serious because the included trials differed substantially in clinical settings, participant health conditions, mandala formats, intervention doses, control conditions, and outcome scales. Overall, the certainty of evidence was judged to be very low for both anxiety symptoms and depressive symptoms (Table 2).

**Table 2:** Summary of findings and certainty of evidence.

Outcome	No. of studies	Participants, *n*	Effect estimate	Heterogeneity	Risk of bias	Inconsistency	Indirectness	Imprecision	Publication bias	Overall certainty
Anxiety symptoms	7 RCTs	531	SMD = −0.95, 95% CI −1.43 to −0.48; *p* < 0.0001	*I*^2^ = 85%	Serious^a^	Very serious^b^	Serious^c^	Serious^d^	Not formally assessed^e^	Very low
Depressive symptoms	4 RCTs	283	SMD = −1.21, 95% CI −1.86 to −0.56; *p* = 0.0002	*I*^2^ = 84%	Serious^a^	Very serious^b^	Serious^c^	Serious^d^	Not formally assessed^e^	Very low

a Risk of bias was downgraded because most included studies were judged as having “some concerns” or “high risk of bias” according to RoB 2. Common limitations included insufficient reporting of randomization procedures, limited information on allocation concealment, lack of blinding of outcome assessment, and potential selective reporting.

b Inconsistency was downgraded because substantial statistical heterogeneity was observed for both anxiety symptoms (*I*^2^ = 85%) and depressive symptoms (*I*^2^ = 84%). The included studies differed in participant characteristics, clinical settings, intervention format, intervention frequency, intervention duration, control conditions, and outcome measures.

c Indirectness was downgraded because the included trials involved clinically diverse populations and settings, including community-dwelling older adults, nursing-home residents, and patients with chronic heart failure, cancer, stroke, or perioperative conditions. For anxiety symptoms, one study assessed death anxiety rather than general anxiety, which further contributed to conceptual indirectness.

d Imprecision was downgraded because the evidence was based on a small number of trials with limited sample sizes. Several subgroup and sensitivity analyses included very few studies, reducing the stability and precision of the estimates.

e Publication bias could not be formally assessed because fewer than 10 studies were available for each outcome. In addition, grey literature sources and clinical trial registries were not systematically searched; therefore, unpublished null findings cannot be ruled out. CI, confidence interval; GRADE, Grading of Recommendations Assessment, Development and Evaluation; *I*^2^, inconsistency statistic; RCT, randomized controlled trial; RoB 2, Cochrane Risk of Bias 2 tool; SMD, standardized mean difference. SMD < 0 favors mandala-based art intervention.

## 4 Discussion

### 4.1 Principal findings

This systematic review and meta-analysis synthesized nine randomized controlled trials evaluating mandala-based art interventions for anxiety and depressive symptoms in middle-aged and older adults. The pooled results suggested that mandala-based interventions were associated with reductions in both anxiety and depressive symptoms relative to control conditions. These findings align with broader evidence indicating that creative arts and visual art-based interventions may support emotional well-being and mental health in clinical, community, and older-adult settings (Stuckey and Nobel, 2010; Fancourt and Finn, 2019; Dunphy et al., 2019; Quinn et al., 2025). Collectively, the present evidence provides preliminary support that mandala-based art activities may serve as a complementary emotional-support strategy for middle-aged and older adults in community, long-term care, and clinical contexts.

However, several caveats warrant attention. Both primary outcomes exhibited substantial heterogeneity, with key sources discussed in a dedicated subsection. Similar heterogeneity has also been noted in broader reviews of arts-based and creative arts interventions, where effects may vary according to setting, delivery mode, facilitator expertise, and participant characteristics (Dunphy et al., 2019; Fancourt and Finn, 2019; Quinn et al., 2025). Furthermore, three studies were judged to have a high risk of bias and six to have some concerns, and the evidence was rated as very low. Therefore, while the findings support potential benefit, they do not establish an optimal clinical protocol nor justify replacing established psychological or medical care.

### 4.2 Sources of heterogeneity and certainty of evidence

Substantial heterogeneity was observed for both anxiety and depressive symptoms, indicating that the pooled estimates should be interpreted with caution. Several sources may explain this variability. First, the included studies enrolled clinically diverse populations, including community-dwelling adults, residents of long-term care facilities, and patients with chronic heart failure, cancer, stroke, or perioperative conditions. These groups may differ in baseline emotional distress, disease burden, care environment, and responsiveness to non-pharmacological interventions. Second, intervention protocols varied in format, frequency, duration, and delivery context, ranging from single-session mandala coloring to repeated drawing or painting interventions. Third, control conditions differed across studies, including usual care, no intervention, reading, health education, or psychological support, which may have influenced the relative effect sizes. Fourth, outcome measures were not fully consistent across studies, and the anxiety analysis included one study assessing death anxiety rather than general anxiety. Although the post-hoc sensitivity analysis excluding this study did not change the overall direction of the effect, the conceptual difference between death anxiety and general anxiety remains an important source of clinical heterogeneity. Despite this diversity, pooling was considered methodologically acceptable because the included studies shared the same broad design and intervention-outcome framework: randomized controlled trials of mandala-based activities targeting anxiety-related or depressive symptoms. Therefore, the meta-analysis should be understood as estimating a broad average effect across heterogeneous settings rather than a precise effect for a specific clinical population or intervention protocol.

In addition to heterogeneity, the certainty of the evidence was very low for both outcomes due to risk-of-bias concerns, inconsistency, indirectness, imprecision, and the small number of trials. Therefore, the present findings should be viewed as preliminary evidence of potential benefit rather than definitive evidence of clinical effectiveness. Larger, better-reported, and methodologically rigorous randomized trials are needed to confirm the magnitude and durability of the effects.

### 4.3 Possible mechanisms

Several mechanisms might explain why mandala-based art interventions could reduce anxiety and depressive symptoms. First, the circular, symmetrical, and repetitive visual structure of mandalas may facilitate attentional focus. Experimental studies of mandala coloring have suggested that structured coloring tasks can reduce anxiety compared with unstructured free drawing in experimental settings, possibly because the repetitive and bounded visual task supports focused attention and emotional containment (Curry and Kasser, 2005; van der Vennet and Serice, 2012). Such focused engagement may reduce repetitive worry, rumination, and excessive attention to illness-related discomfort. For individuals experiencing anxiety, this attentional redirection could be particularly relevant because anxiety is often sustained by heightened vigilance and physiological arousal.

Second, mandala activity may provide a non-verbal channel for emotional expression. Some middle-aged and older adults may find it difficult to articulate distress verbally, especially in medical or institutional settings. Color selection, pattern construction, and visual composition can allow participants to express emotion indirectly, organize internal experience, and cultivate a sense of psychological containment. This explanation aligns with broader accounts of visual art-making as a process that supports self-expression, meaning-making, and affect regulation (Stuckey and Nobel, 2010; Fancourt and Finn, 2019; Dunphy et al., 2019). Third, completing a tangible artwork may enhance perceived control, mastery, and a sense of accomplishment. Visual art-making has also been associated with reductions in physiological stress markers such as cortisol, suggesting that creative engagement may involve both psychological and physiological stress-regulation pathways (Kaimal et al., 2016). These experiences could be particularly meaningful for individuals with depressive symptoms, who often experience diminished motivation, low self-worth, and reduced pleasure. These mechanisms offer plausible explanations for why mandala-based art interventions may support emotional regulation, but the present review cannot determine whether intervention format or frequency modifies the magnitude of the effects.

### 4.4 Interpretation of subgroup findings

Exploratory subgroup analyses were conducted only to describe whether effect estimates varied across intervention frequency and mandala format categories. For anxiety symptoms, the pooled estimate was numerically different between single-session and repeated-intervention subgroups. This numerical difference should not be interpreted as evidence of a dose–response effect because the subgroup comparison was indirect, underpowered, and confounded by population, intervention duration, control condition, and study quality. Importantly, our subgroup analyses are limited by very small numbers of studies per subgroup (often 2 or 3) and by confounding among mandala format, population, and study design. Therefore, these findings should be considered hypothesis-generating only, and no conclusion about the comparative effectiveness of different mandala formats or intervention frequencies is warranted at this time. Although repeated exposure could theoretically provide more opportunities for practice and emotional regulation, the present subgroup data cannot test this explanation. Experimental evidence from mandala coloring and adult coloring studies has examined both brief coloring activities and repeated practice (Curry and Kasser, 2005; van der Vennet and Serice, 2012; Flett et al., 2017), but these studies do not establish an optimal intervention frequency for middle-aged and older adults.

For depressive symptoms, pooled estimates varied numerically across frequency subgroups, but these variations should be treated as descriptive because subgroup membership overlapped with differences in population, baseline symptom severity, disease context, and intervention duration. Likewise, the numerical estimates differed between open-ended mandala drawing or painting and structured coloring; however, these differences should not be interpreted as evidence of comparative superiority. Open-ended formats and structured coloring differ conceptually in the degree of personal expression and standardization (Dunphy et al., 2019; Fancourt and Finn, 2019), but the present subgroup analyses cannot determine whether these differences translate into different clinical effects. Therefore, direct randomized comparative trials that hold population, control condition, intervention duration, and study quality more constant are needed before any recommendation can be made regarding optimal mandala format or intervention frequency.

### 4.5 Clinical and public health implications

Mandala-based art interventions may be considered as low-cost, low-risk, and easily deliverable complementary emotional-support activities in community centers, nursing homes, rehabilitation units, oncology care, and chronic disease management settings. These activities require minimal equipment, can be implemented individually or in groups, and may be facilitated by nurses, rehabilitation therapists, mental health workers, or trained volunteers. Such features are particularly valuable in settings where access to specialized mental health services is limited. The potential value of accessible arts-based interventions is supported by broader arts-and-health evidence and by recent findings that group arts interventions can reduce depression and anxiety in older adults, especially in care-home contexts (Fancourt and Finn, 2019; Ciasca et al., 2018; Quinn et al., 2025).

However, the findings should not be interpreted as evidence that mandala-based art interventions can replace psychotherapy, medication, or other evidence-based mental health treatments. Rather, they may be considered as adjunctive emotional-support activities for individuals with mild to moderate symptoms or as part of broader psychosocial care. This interpretation is consistent with evidence that arts-based interventions are often most appropriately positioned as complementary approaches within multidisciplinary care rather than as stand-alone replacements for established treatments (Stuckey and Nobel, 2010; Ciasca et al., 2018; Fancourt and Finn, 2019). Clear protocols, facilitator training, monitoring of adverse experiences, and referral pathways for participants with severe or worsening symptoms would be needed before widespread implementation. Successful implementation requires attention to several factors. First, facilitators (e.g., nurses, activity coordinators, trained volunteers) need basic instruction on how to introduce the activity, provide a supportive environment, and avoid over-interpretation of participants’ artwork. Second, clear protocols should specify session frequency and duration, and whether the activity is structured (e.g., pre-drawn mandalas) or open-ended. Third, and most critically, implementation sites should establish referral pathways to mental health professionals for participants who show signs of worsening anxiety, depression, or emotional distress during or after the intervention. Mandala activities should be positioned as a complementary psychosocial support, not as a substitute for psychotherapy or pharmacotherapy. With these safeguards, mandala-based interventions can be a valuable addition to the supportive care toolkit for middle-aged and older adults.

### 4.6 Strengths and limitations

This review has several strengths. It focused specifically on randomized controlled trials, included both English and Chinese databases, examined both anxiety and depressive symptoms, employed random-effects meta-analysis to account for expected heterogeneity, and incorporated risk-of-bias and certainty-of-evidence assessments. The review also treated subgroup findings as exploratory, which is appropriate given the limited number of studies.

Several limitations should be acknowledged. First, although the protocol was registered in PROSPERO, some methodological details, such as subgroup analyses and certainty assessment, may have been refined during the review process. Second, while multiple English and Chinese databases were searched, trial registries and grey literature sources were not systematically examined; therefore, unpublished or ongoing trials may have been missed. Third, the methodological quality of the included studies was suboptimal. Six studies had “some concerns,” and three had “high risk of bias” according to RoB 2. Common limitations included insufficient detail on randomization procedures, lack of allocation concealment, and absence of blinded outcome assessment. These limitations likely lead to an overestimation of the true intervention effects. Consequently, our pooled effect sizes (SMD = −0.95 for anxiety and −1.21 for depression) should be interpreted as provisional best-case estimates; the actual effects in real-world settings may be more modest. This concern was central to our decision to rate the certainty of evidence as very low (see Table 2). Fourth, conceptual heterogeneity may have arisen from the inclusion of death anxiety (Dincer et al., 2026) together with general anxiety measures (STAI, SAS, HAMA). Although the post-hoc sensitivity analysis excluding this study did not change the statistical significance of the pooled effect, death anxiety is conceptually distinct from general anxiety. Therefore, the anxiety findings should be interpreted with caution. Fifth, formal publication-bias tests could not be conducted because fewer than 10 studies were available for each outcome. Therefore, we cannot rule out the possibility that small unpublished studies with null results exist, which could attenuate the observed effect sizes. Finally, the evidence base was small and included studies with some concerns or high risk of bias, limiting confidence in the pooled estimates.

### 4.7 Future research

Future trials should employ adequately powered randomized designs, clear allocation concealment, blinded outcome assessment where feasible, pre-registered protocols, and standardized reporting of intervention components. Studies should clearly describe mandala format, session duration, facilitator qualifications, materials, instructions, adherence, and co-interventions. Standardized reporting is especially important because previous reviews of arts-based interventions have highlighted wide variability in intervention content, delivery process, and outcome assessment (Dunphy et al., 2019; Fancourt and Finn, 2019; Quinn et al., 2025). Longer-term follow-up would be necessary to determine whether emotional benefits persist beyond the immediate intervention period. Future trials should also prioritize transparency in intervention reporting. We recommend adherence to the TIDieR checklist (Template for Intervention Description and Replication). Specifically, studies should describe: (a) the qualifications, training, and background of the facilitator(s); (b) a detailed session-by-session protocol or script, including instructions given to participants; (c) how adherence and participant engagement were monitored (e.g., session attendance, completion of mandala activities, and any adverse events or emotional distress); (d) the intervention setting (individual vs. group, and the physical environment); and (e) any co-interventions or concomitant treatments. Without such details, it remains unclear which specific components of mandala-based interventions drive the observed effects, and replication efforts will be hindered.

Future research should also directly compare structured mandala coloring with open-ended mandala drawing or painting, examine dose–response relationships, and evaluate whether effects differ by setting, baseline symptom severity, health condition, and cognitive status. In addition to symptom scales, subsequent studies may consider outcomes such as quality of life, sleep, loneliness, perceived control, intervention acceptability, adverse experiences, and physiological indicators of stress regulation (Kaimal et al., 2016; Dunphy et al., 2019; Quinn et al., 2025).

## 5 Conclusion

The current very low-certainty evidence suggests a potential association between mandala-based art interventions and reductions in anxiety and depressive symptoms among middle-aged and older adults. These interventions are simple, low-cost, and potentially suitable for community, long-term care, and clinical contexts. However, the existing evidence is constrained by substantial heterogeneity, limited sample sizes, and concerns about risk of bias. The available evidence should therefore be considered preliminary. More rigorous, adequately powered, and transparently reported randomized controlled trials are needed to determine whether the observed effects are reproducible and clinically meaningful, identify the most suitable intervention format and dose, and examine whether benefits are sustained over time.

## Data Availability

The original contributions presented in the study are included in the article/Supplementary material, further inquiries can be directed to the corresponding author.
