# Supplementary material for: Mandala-based art interventions for anxiety and depressive symptoms in middle-aged and older adults: a systematic review and meta-analysis
Source: Front Psychol. 2026 Jul 1;17:1877910. doi: 10.3389/fpsyg.2026.1877910 (PMC13368728; doi:10.3389/fpsyg.2026.1877910)
Supplement: Supplementary file 1 [file Table_1.DOCX]

Supplementary Material

## Table S1. Full search strategies

| **Database** | **Search Strategy** |
| --- | --- |
| PubMed | Primary search:  ("Mandala"[Title/Abstract] OR "Mandala therapy"[Title/Abstract] OR "Mandala art therapy"[Title/Abstract] OR "Mandala art"[Title/Abstract] OR "Mandala painting"[Title/Abstract] OR "Mandala drawing"[Title/Abstract] OR "Mandala coloring"[Title/Abstract] OR "Mandala colouring"[Title/Abstract] OR "Art Therapy"[Mesh] OR "art therapy"[Title/Abstract] OR "painting therapy"[Title/Abstract])  AND  ("Aged"[Mesh] OR "Aged, 80 and over"[Mesh] OR "Middle Aged"[Mesh] OR "Older Adult*"[Title/Abstract] OR "Elderly"[Title/Abstract] OR "Senior*"[Title/Abstract] OR "Geriatric*"[Title/Abstract] OR "Aging"[Mesh] OR middle-aged[Title/Abstract] OR "middle age"[Title/Abstract] OR "middle-aged adult*"[Title/Abstract])  AND  ("Anxiety"[Mesh] OR "Anxiety"[Title/Abstract] OR "Anxiety Disorders"[Mesh] OR "Depression"[Mesh] OR "Depression"[Title/Abstract] OR "Depressive Disorder"[Mesh] OR "depressive symptom*"[Title/Abstract] OR "Stress, Psychological"[Mesh] OR "Psychological Distress"[Title/Abstract] OR "Mental Health"[Mesh] OR "Quality of Life"[Mesh] OR "Well-Being"[Title/Abstract])  AND  ("randomized controlled trial"[Publication Type] OR "controlled clinical trial"[Publication Type] OR randomized[Title/Abstract] OR randomised[Title/Abstract] OR randomly[Title/Abstract] OR RCT[Title/Abstract] OR placebo[Title/Abstract] OR "clinical trial"[Title/Abstract])  NOT ("animals"[Mesh] NOT "humans"[Mesh])  Supplementary search without RCT-related terms:  ("Mandala"[Title/Abstract] OR "Mandala therapy"[Title/Abstract] OR "Mandala art therapy"[Title/Abstract] OR "Mandala art"[Title/Abstract] OR "Mandala painting"[Title/Abstract] OR "Mandala drawing"[Title/Abstract] OR "Mandala coloring"[Title/Abstract] OR "Mandala colouring"[Title/Abstract] OR "Art Therapy"[Mesh] OR "art therapy"[Title/Abstract] OR "painting therapy"[Title/Abstract])  AND  ("Aged"[Mesh] OR "Aged, 80 and over"[Mesh] OR "Middle Aged"[Mesh] OR "Older Adult*"[Title/Abstract] OR "Elderly"[Title/Abstract] OR "Senior*"[Title/Abstract] OR "Geriatric*"[Title/Abstract] OR "Aging"[Mesh] OR middle-aged[Title/Abstract] OR "middle age"[Title/Abstract] OR "middle-aged adult*"[Title/Abstract])  AND  ("Anxiety"[Mesh] OR "Anxiety"[Title/Abstract] OR "Anxiety Disorders"[Mesh] OR "Depression"[Mesh] OR "Depression"[Title/Abstract] OR "Depressive Disorder"[Mesh] OR "depressive symptom*"[Title/Abstract] OR "Stress, Psychological"[Mesh] OR "Psychological Distress"[Title/Abstract] OR "Mental Health"[Mesh] OR "Quality of Life"[Mesh] OR "Well-Being"[Title/Abstract])  NOT ("animals"[Mesh] NOT "humans"[Mesh]) |
| Wanfang | Primary search:  主题:(曼陀罗 OR 曼陀罗绘画 OR 曼陀罗涂色 OR 曼陀罗彩绘 OR 曼陀罗艺术 OR 曼陀罗艺术治疗 OR 绘画治疗 OR 艺术治疗)  AND  主题:(老年人 OR 老年 OR 老人 OR 长者 OR 中年 OR 中老年 OR 55岁以上)  AND  主题:(焦虑 OR 焦虑症状 OR 抑郁 OR 抑郁症状 OR 心理健康 OR 心理疗愈 OR 心理痛苦 OR 生活质量)  AND  主题:(随机 OR 随机对照 OR 随机分组 OR RCT OR 临床试验)  Supplementary search without RCT-related terms:  主题:(曼陀罗 OR 曼陀罗绘画 OR 曼陀罗涂色 OR 曼陀罗彩绘 OR 曼陀罗艺术 OR 曼陀罗艺术治疗 OR 绘画治疗 OR 艺术治疗)  AND  主题:(老年人 OR 老年 OR 老人 OR 长者 OR 中年 OR 中老年 OR 55岁以上)  AND  主题:(焦虑 OR 焦虑症状 OR 抑郁 OR 抑郁症状 OR 心理健康 OR 心理疗愈 OR 心理痛苦 OR 生活质量) |
| Web of Science | Primary search:  #1 TS=(mandala* OR "mandala therapy" OR "mandala art therapy" OR "mandala art" OR "mandala painting" OR "mandala drawing" OR "mandala coloring" OR "mandala colouring" OR "art therapy" OR "painting therapy")  #2 TS=("older adult*" OR elderly OR senior* OR geriatric* OR aging OR aged OR middle-aged OR "middle age" OR "middle-aged adult*" OR "Middle Aged")  #3 TS=(anxiety OR "anxiety disorder*" OR depression OR "depressive disorder*" OR "depressive symptom*" OR "psychological distress" OR "mental health" OR "quality of life" OR QoL OR well-being)  #4 TS=("randomized controlled trial" OR "randomised controlled trial" OR RCT OR randomized OR randomised OR randomly OR "clinical trial" OR placebo)  #5 #1 AND #2 AND #3 AND #4  Supplementary search without RCT-related terms:  #6 #1 AND #2 AND #3 |
| Embase | Primary search:  ('mandala'/exp OR mandala*,ab,kw OR 'mandala therapy',ab,kw OR 'mandala art therapy',ab,kw OR 'mandala art',ab,kw OR 'mandala painting',ab,kw OR 'mandala drawing',ab,kw OR 'mandala coloring',ab,kw OR 'mandala colouring',ab,kw OR 'art therapy'/exp OR 'art therapy',ab,kw OR 'painting therapy',ab,kw)  AND  ('aged'/exp OR 'middle aged'/exp OR aged,ab,kw OR elderly,ab,kw OR 'older adult*',ab,kw OR senior*,ab,kw OR geriatric*,ab,kw OR 'middle-aged*',ab,kw OR 'middle age*',ab,kw OR 'middle-aged adult*',ab,kw)  AND  ('anxiety'/exp OR anxiety,ab,kw OR 'anxiety disorder'/exp OR 'anxiety disorder*',ab,kw OR 'depression'/exp OR depression,ab,kw OR 'depressive disorder'/exp OR 'depressive symptom*',ab,kw OR 'mental health'/exp OR 'mental health',ab,kw OR 'psychological distress',ab,kw OR 'quality of life'/exp OR 'quality of life',ab,kw OR well-being,ab,kw)  AND  ('randomized controlled trial'/exp OR 'randomized controlled trial',ab,kw OR 'randomised controlled trial',ab,kw OR rct,ab,kw OR randomized,ab,kw OR randomised,ab,kw OR randomly,ab,kw OR 'controlled clinical trial',ab,kw OR 'clinical trial',ab,kw OR placebo,ab,kw)  Supplementary search without RCT-related terms:  ('mandala'/exp OR mandala*,ab,kw OR 'mandala therapy',ab,kw OR 'mandala art therapy',ab,kw OR 'mandala art',ab,kw OR 'mandala painting',ab,kw OR 'mandala drawing',ab,kw OR 'mandala coloring',ab,kw OR 'mandala colouring',ab,kw OR 'art therapy'/exp OR 'art therapy',ab,kw OR 'painting therapy',ab,kw)  AND  ('aged'/exp OR 'middle aged'/exp OR aged,ab,kw OR elderly,ab,kw OR 'older adult*',ab,kw OR senior*,ab,kw OR geriatric*,ab,kw OR 'middle-aged*',ab,kw OR 'middle age*',ab,kw OR 'middle-aged adult*',ab,kw)  AND  ('anxiety'/exp OR anxiety,ab,kw OR 'anxiety disorder'/exp OR 'anxiety disorder*',ab,kw OR 'depression'/exp OR depression,ab,kw OR 'depressive disorder'/exp OR 'depressive symptom*',ab,kw OR 'mental health'/exp OR 'mental health',ab,kw OR 'psychological distress',ab,kw OR 'quality of life'/exp OR 'quality of life',ab,kw OR well-being,ab,kw) |
| SinoMed | Primary search:  #1 (曼陀罗 OR 曼陀罗绘画 OR 曼陀罗涂色 OR 曼陀罗彩绘 OR 曼陀罗艺术 OR 曼陀罗艺术治疗 OR 绘画治疗 OR 艺术治疗)  #2 (老年人 OR 老年 OR 老人 OR 长者 OR 中年 OR 中老年 OR 55岁以上)  #3 (焦虑 OR 焦虑症状 OR 抑郁 OR 抑郁症状 OR 心理健康 OR 心理痛苦 OR 生活质量)  #4 (随机 OR 随机对照 OR 随机分组 OR RCT OR 临床试验)  #5 #1 AND #2 AND #3 AND #4  Supplementary search without RCT-related terms:  #6 #1 AND #2 AND #3 |

Note. The primary searches included RCT-related terms to improve specificity and screening manageability. Because such terms may reduce sensitivity in arts-based intervention research, supplementary searches without the RCT-related search block were additionally conducted in each database. These supplementary searches used only intervention-, population-, and outcome-related terms. The supplementary searches identified additional records for screening but did not identify additional eligible randomized controlled trials. The final number of records identified from the updated database searches was 709, consistent with the PRISMA flow diagram.
